# Structural aspects of nucleotide ligand binding by a bacterial 2H phosphoesterase

**DOI:** 10.1371/journal.pone.0170355

**Published:** 2017-01-31

**Authors:** Matti Myllykoski, Petri Kursula

**Affiliations:** 1 Faculty of Biochemistry and Molecular Medicine & Biocenter Oulu, University of Oulu, Oulu, Finland; 2 Department of Biomedicine, University of Bergen, Bergen, Norway; Russian Academy of Medical Sciences, RUSSIAN FEDERATION

## Abstract

The 2H phosphoesterase family contains enzymes with two His-X-Ser/Thr motifs in the active site. 2H enzymes are found in all kingdoms of life, sharing little sequence identity despite the conserved overall fold and active site. For many 2H enzymes, the physiological function is unknown. Here, we studied the structure of the 2H family member LigT from *Escherichia coli* both in the apo form and complexed with different active-site ligands, including ATP, 2′-AMP, 3′-AMP, phosphate, and NADP^+^. Comparisons to the well-characterized vertebrate myelin enzyme 2′,3′-cyclic nucleotide 3′-phosphodiesterase (CNPase) highlight specific features of the catalytic cycle and substrate recognition in both enzymes. The role played by the helix α7, unique to CNPases within the 2H family, is apparently taken over by Arg130 in the bacterial enzyme. Other residues and loops lining the active site groove are likely to be important for RNA substrate binding. We visualized conformational changes related to ligand binding, as well as the position of the nucleophilic water molecule. We also present a low-resolution model of *E*. *coli* LigT bound to tRNA in solution, and provide a model for RNA binding by LigT, involving flexible loops lining the active site cavity. Taken together, our results both aid in understanding the common features of 2H family enzymes and help highlight the distinct features in the 2H family members, which must result in different reaction mechanisms. Unique aspects in different 2H family members can be observed in ligand recognition and binding, and in the coordination of the nucleophilic water molecule and the reactive phosphate moiety.

## Introduction

The 2H phosphoesterase superfamily is an ancient group of proteins and protein domains characterized by a common fold and a few conserved active site residues [1,2]. Various catalytic activities have been assigned for the 2H enzymes: hydrolysis of a 2′,3′-cyclic phosphate, in either nucleotides or 3′-ends of RNA molecules, into 2′-phosphate [3–5], hydrolysis of ADP-ribose 1′′,2′′-cyclic phosphates into ADP-ribose 1′′-phosphate [6–8], generation and/or cleavage of 2′-5′-linkages between nucleotides or RNA molecules, such as tRNA halves [3,9–13], and 3′-5′ exonucleolytic removal of terminal uridine nucleotides from snRNA with the simultaneous generation of 2′,3′-cyclic phosphates at the terminus [14]. The structurally best-characterized 2H enzyme is the mammalian myelin enzyme 2′,3′-cyclic nucleotide 3′-phosphodiesterase (CNPase) [15–19], but even for this enzyme, the biological function remains enigmatic [5].

*E*. *coli* LigT is a 20-kDa protein that exhibits 2′,3′-cyclic nucleotide 3′-phosphodiesterase and 2′-5′-ligase/phosphodiesterase activities [3,9], but the biological function of the enzyme is unknown. It is potentially a source of 2′-5′ oligoadenylates (2-5A) and similar compunds with 2′-5′-linkage detected in *E*. *coli* [20]. A genomic knockout of LigT in *E*. *coli* did not obviously affect cellular growth or viability in laboratory conditions, while LigT overexpression produced a phenotype sensitive to elevated temperature [9]. A LigT orthologue in *Deinococcus radiodurans* was massively upregulated after acute irradiation, and it was speculated to function in the handling of damaged RNA species [21].

In a recent study, *E*. *coli* LigT was crystallized, and its structure was refined in complex with the *in vitro* reaction product 2′-AMP [22]. We extend the previous study here, providing high-resolution crystallographic data and different active-site ligand complexes. Comparisons to the apo form of the enzyme determined in the current work allow the highlighting of conformational changes and binding determinants of the reactive species. Based on further comparisons to mouse CNPase, we also modelled the substrate complex of LigT and discuss unique features of each protein. An extended binding surface for RNA substrates is also identified, which possibly involves flexible loops of the 2H family. All in all, we provide novel data on the structure-function relationships in a bacterial 2H enzyme, which can be used to understand the common and divergent properties of enzymes in the entire 2H superfamily.

## Results and discussion

### Overall structure

The structure of the putative tRNA ligase LigT from *E*. *coli* was determined by X-ray crystallography in the apo form. In addition, a number of LigT complex structures with active-site ligands were solved, hence significantly extending the earlier results on the LigT structure, which was only available as a complex with the reaction product 2′-AMP [22]. Intriguingly, a total of five different crystal forms were observed in this study, which also highlights the flexible properties of the enzyme.

**Fig 1:**
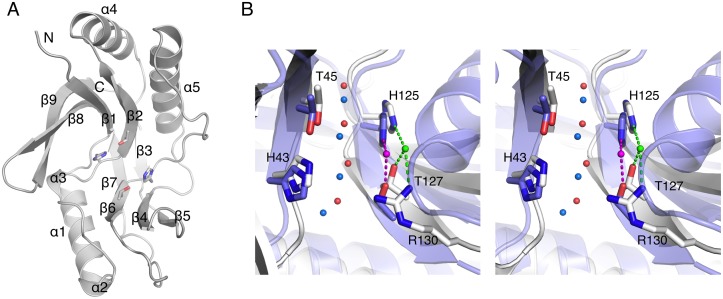
The structure of *E*. *coli* LigT. A. Overall structure of LigT. Secondary structure elements and the N and C termini are labeled, and the two active site HxT motif side chains are also shown. B. Stereo view of the organization and conservation of the active site between LigT (white) and mouse CNPase (blue) [18]. The four water molecules between strands 2 and 6 are conserved (LigT, red; CNPase, blue). The nucleophilic water molecule in LigT (green) is coordinated by His125 and Arg130, while the corresponding water molecule in CNPase (magenta) interacts with the N terminus of helix 7 (right) and the catalytic histidine.

The LigT structure presents a typical 2H phosphoesterase fold, in which the catalytic residues reside at the beginning of strands β2 and β6 (Fig 1A). As in other 2H family members, the active site has 2-fold symmetry; this symmetry also includes the 4 water molecules at the bottom of the active site, connecting the active-site β strands through water-mediated hydrogen bonds (Fig 1B). These water molecules coordinate the substrate/product throughout the reaction in CNPase [17,18], and they are likely to play a similar role in LigT and other 2H phosphoesterases. The strict conservation of the active-site water structure between 2H enzymes is remarkable, taking into account the amino acid sequence identity of slightly above 10% between CNPase and LigT.

### Liganded complexes

**Fig 2:**
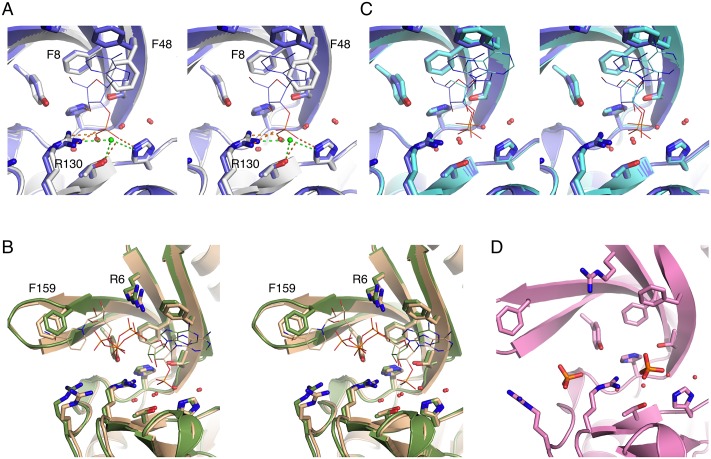
Crystal structures of LigT complexed with different active-site ligands. A. Comparison between apo LigT (white) and the 2′-AMP complex (blue). Interactions of the ligand phosphate group are shown as orange dashed lines and those of the nucleophilic water molecule (green) in the apo structure as green dashed lines. B. Complexes with NADP^+^ (light brown) and ATP (green). Note the stacking of the NADP^+^ nicotinamide ring against Phe159 and the coordination of the 5′-phosphate in both ligands by Arg6. C. Binding modes of 2′-AMP (blue) and 3′-AMP (light blue). The same recognition elements are at play, but especially the ribose ring binds differently. D. Cocrystallization with tRNA resulted in a complex with two phosphate ions, one in the active site and the other one in a nearby pocket surrounded by Arg residues.

For an insight into the active-site properties and catalytic mechanism, we co-crystallized LigT with different nucleotide compounds in the active site (Table 1, Fig 2), including substrates, products, and other compounds. In addition to the presumed obvious substrates and products, we also used other nucleotide compounds in an attempt to screen LigT crystallographically for nucleotide-like ligand-binding properties. This was considered of interest, as open questions remain related to the physiological function of both LigT and many 2H family enzymes in general. All structures had more than one protein chain in the asymmetric unit, and in general, the one with best-defined electron density for ligands was used in the analyses below.

**Table 1:** Crystallographic data collection and structure refinement statistics.

Ligand	none	phosphate (tRNA)	ATP	2'-AMP	3'-AMP	ATP	NADP^+^
Data collection statistics							
Space group	P2_1_	P1	C2	P42_1_2	P2_1_	C2	C2
Cell parameters							
a (Å)	63.39	54.73	101.95	108.07	63.41	102.15	86.74
b (Å)	88.32	63.52	73.33	108.07	88.35	73.47	91.62
c (Å)	73.84	67.37	77.49	72.64	73.97	77.61	120.61
**α** (°)	90	106.26	90	90	90	90	90
**β** (°)	115.01	106.14	128.54	90	114.95	128.34	102.32
**γ** (°)	90	103.06	90	90	90	90	90
No. of molecules in ASU	4	4	2	2	4	2	4
Wavelength (Å)	1.03	1.04	1.04	1.04	1.04	1.04	1.03
High resolution (Å)	2.10	2.80	2.10	2.46	2.75	1.80	1.70
High-resolution shell (Å)	2.15–2.10	2.88–2.80	2.15–2.10	2.52–2.46	2.82–2.75	1.85–1.80	1.74–1.70
Unique reflections	43063 (3154)	18651 (1460)	26124 (1890)	15451 (613)	19216 (1356)	41617 (3088)	100969 (7397)
Multiplicity	3.81 (3.83)	2.21 (2.20)	3.81 (3.78)	6.60 (2.58)	3.80 (3.76)	3.38 (3.33)	3.80 (3.86)
Completeness (%)	99.6 (99.8)	95.7 (94.3)	99.2 (99.2)	95.4 (52.4)	99.3 (97.8)	99.2 (99.5)	99.8 (99.8)
R_merge_	0.076 (0.918)	0.126 (0.942)	0.075 (0.829)	0.079 (0.621)	0.092 (0.804)	0.047 (1.061)	0.053 (1.043)
R_meas_	0.089 (1.067)	0.171 (1.270)	0.088 (0.967)	0.086 (0.747)	0.107 (0.939)	0.056 (1.258)	0.061 (1.211)
<I/**σ**(I)>	11.93 (1.30)	7.21 (1.02)	14.16 (1.65)	18.01 (1.51)	14.11 (1.76)	15.47 (1.31)	13.92 (1.23)
CC_½_ (%)	99.8 (58.1)	97.8 (41.3)	99.8 (64.8)	99.9 (71.2)	99.6 (62.9)	99.9 (50.8)	99.9 (61.9)
Wilson B (Å^2^)	35.6	42.6	32.0	47.4	46.1	28.8	27.3
Refinement statistics							
Resolution range (Å)	36.60–2.10	22.96–2.80	23.37–2.10	29.04–2.46	28.75–2.75	19.70–1.8	117.84–1.70
High-resolution shell (Å)	2.15–2.10	2.88–2.80	2.15–2.10	2.54–2.46	2.82–2.75	1.85–1.80	1.74–1.70
Reflections in the working set	40878 (2698)	16784 (1279)	24120 (1685)	13888 (728)	17272 (1183)	39641 (2810)	98860 (6928)
Reflections in the test set	2153 (142)	1859 (144)	1997 (141)	1544 (78)	1922 (126)	1975 (147)	1998 (139)
R value (%)	17.74 (27.52)	21.77 (34.36)	20.18 (26.83)	19.70 (34.24)	21.17 (31.25)	17.06 (29.33)	18.34 (38.32)
R_free_ value (%)	22.67 (29.43)	25.80 (40.78)	24.76 (32.97)	25.28 (39.44)	25.87 (38.53)	20.35 (34.02)	21.26 (41.66)
RMSD bond length (Å)	0.004	0.003	0.008	0.005	0.003	0.019	0.007
RMSD angle (°)	0.981	0.705	1.148	0.937	0.830	1.834	1.321
Atoms in ASU*	11652 (5579)	5490	2949	5771 (2854)	5475	3322 (12)	7048
Protein atoms in ASU	5561	5444	2721	2807	5449	2902	6059
Ligand atoms in ASU	3	25	31	47	23	99	351
Water molecules in ASU	509	21	197	63	3	279	638
Mean B value (Å^2^)	54.9	60.80	36.45	64.03	48.21	42.91	39.45
Ramachandran plot							
Favoured regions (%)	99.26	99.40	97.88	98.25	97.74	99.72	98.63
Allowed regions (%)	0.59	0.60	2.12	1.75	1.36	0.28	1.09
Outlier residues (%)	0.15	0	0	0	0.90	0	0.27
Clashscore	2.42	3.93	5.09	2.46	5.02	12.07	8.46
Rotamer outliers (%)	0.87	1.79	6.07	3.03	0.89	1.33	2.23
**PDB entry**	5ldi	5ldj	5ldk	5ldm	5ldo	5ldp	5ldq

* Number of riding hydrogen atoms is shown in parentheses.

Previously, LigT has been crystallized with the reaction product 2′-AMP [22]. We also observed such a complex after cocrystallization with 2′,3′-cAMP (Fig 2A). The binding mode is similar to that seen before, and the 2′-AMP ligand is well defined in one of the two LigT monomers, while the occupancy in the other monomer is low. Binding involves aromatic stacking of the nucleotide base against Phe48 and a C-H…π interaction between the ribose ring and Phe8. In addition to the catalytic residues, Arg130 plays a key role in coordinating the phospho moiety of the product. Considering reaction geometry, a water molecule coordinated by His125 in the apo structure can now be designated as the nucleophilic water (Fig 2A), analogously to CNPase. The complex also proves that LigT has CNPase activity towards 2′,3′-cyclic mononucleotides, since the reaction product is bound in the crystals grown in the presence of the substrate.

A possible role for 2′,3′-cyclic nucleotides in mammalian systems has been lately discovered [23], but their importance in prokaryotic systems is not known. However, 2′,3′-cyclic cytidine and uridine monophosphate were recently detected in the extracts of *Pseudomonas fluorescens* [24], and several bacterial phosphodiesterases that cleave 2′,3′-cyclic nucleotides have been described over the years [25,26]. Whether LigT activity plays a role in the metabolism of such compounds *in vivo*, remains to be studied.

Another product analogue of the reaction catalysed by LigT, NADP^+^, was also trapped in the active site, being well defined in electron density (Fig 2B). In the NADP^+^ complex, there are 4 monomers in the asymmetric unit, and the nicotinamide end of the ligand binds to each of them differently, having different conformations or being disordered—depending on crystal contacts. The adenosine 2′,5′-bisphosphate moiety in each monomer binds identically, however, and in a mode highly similar to that seen in 2′-AMP. The 5′-phosphate mimics the next phosphodiester in an RNA molecule, and it can be used to deduce further binding determinants for RNA substrates. This phosphate moiety is bound by Arg6 from the N-terminal strand β1 in the crystal structure, and it is likely that this residue plays a direct role in RNA substrate binding also. An additional NADP^+^ fragment is seen stacked on top of Trp82 in one monomer.

We also cocrystallized the enzyme with ATP and 3′-AMP, which are neither substrates nor products. The fact that they bind the active site can imply that they may be weak inhibitors, and it can be taken as evidence of a general propensity to bind nucleotides in the active site. The LigT orthologue protein PF0027 from *Pyrococcus furiosus* was shown to require a GTP cofactor for the RNA ligation reaction [3], while for the *E*. *coli* enzyme, such a cofactor has not been reported. Although we attempted crystallization in the presence of GTP as with ATP, no corresponding GTP electron density was found in the resulting crystals (data not shown). Two similar datasets were collected with ATP, one of which has one ATP bound to only one of the 2 monomers in the asymmetric unit. This ATP molecule probably has a partial occupancy and/or some degree of flexibility, as evidenced by residual difference electron density. The major conformation was built in the structure. The other complex has three ATP molecules for two monomers; the space group remains the same. The reason for this fortuituous ambiguity is unknown. In addition to the ligand in both active sites (Fig 2B), another ATP in the latter crystal form is bound in the vicinity of it in one monomer; this ATP is also involved in crystal contacts. The binding of this second ATP is a strong indication of a propensity for binding further nucleotides in the active site vicinity by *E*. *coli* LigT. Residues interacting with the second ATP include Phe159 and Arg6. Again, the α-5′-phosphate of the active-site ATP is in the same location as the corresponding phosphate in NADP^+^, and it is similarly coordinated by Arg6, highlighting putative RNA recognition features extending from the active site.

As far as the 3′-AMP experiment is concerned, one of the 4 monomers in the asymmetric unit has a bound 3′-AMP molecule, with the phosphate group in the same position as in the 2′-AMP product complex. Binding of the ligand to the remaining monomers is apparently prevented by crystal contacts. The conformation of 3′-AMP differs slightly from that observed for 2′-AMP, but the essential recognition features remain the same; stacking of the base against Phe48, binding of the phosphate to the two HxTx motifs in the active site, and C-H…π interaction of the sugar ring against Phe8 (Fig 2C). As incubation with 2′,3′-cAMP resulted in 2′-AMP, the 3′-AMP complex is irrelevant for the reaction mechanism. To generate 3′-AMP in the LigT reaction, the nucleophilic water should attack from the opposite side.

In addition, attempts to cocrystallize LigT with tRNA resulted in a structure with apparent phosphate ions bound to the active site; these probably originated as impurities in the tRNA preparation. Two distinct PO_4_-binding sites are observed in the active site, on both sides of Arg130 (Fig 2D). Their locations could correspond to binding sites for phosphomoieties in a bound RNA substrate. One of the phosphates lies in the active site, while the second is in a nearby cavity, close to Arg35. The density for the second phosphate molecule was strongest in monomer D, and it was not built into the other three monomers in the model, as the electron density suggested only partial occupancy.

### Substrate and product binding

**Fig 3:**
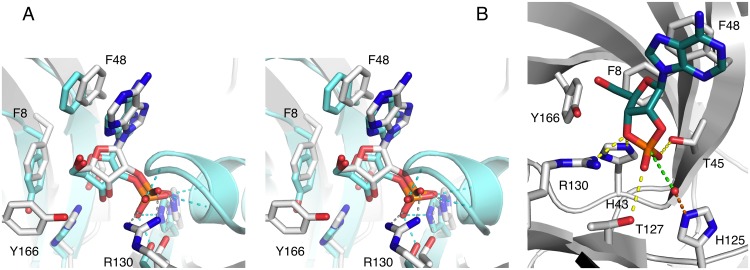
Product and substrate binding. A. Comparison of the binding modes of 2′-AMP in LigT (white) and mouse CNPase (blue). Interactions of the phosphate are indicated by dashed lines; note the binding of the phosphate by Arg130 in LigT, while the phosphate is coordinated to the helix α7 N terminus in CNPase. The other interactions are essentially identical. B. Modelling of the substrate complex of LigT. 2′,3′-cAMP and the nucleophilic water were modeled into the LigT active site based on CNPase structures and the 2′-AMP binding mode to LigT. Polar interactions are shown by yellow dashed lines, and the activation of the water molecule by His125 by an orange dashed line. The direction of nucleophilic attack is shown by the green dashed line. His43 (behind) donates a proton to the leaving group, the ribose 3′-oxygen.

The crystal structure of LigT with 2′-AMP was superimposed on the corresponding complex of CNPase, in order to distinguish common and divergent ligand binding determinants in 2H enzymes. The binding mode of the reaction product is very similar in both enzymes, and the base and sugar moieties make similar interactions. While in CNPase, the N terminus of helix α7 is important in coordinating the reaction product [17], Arg130 is an important residue for binding the corresponding phosphate group in LigT (Fig 3A).

As the crystals obtained in the presence of substrate resulted in the structure of a product complex, we also modeled the likely substrate complex of LigT and 2′,3′-cAMP, based on our earlier liganded complexes of CNPase [16–18], coupled to manual docking and energy minimization. The predicted binding mode and interactions are very similar to those seen in CNPase. Compared to the product binding mode, His125 is not directly interacting with the substrate, but coordinates the nucleophilic water instead (Fig 3B).

**Fig 4:**
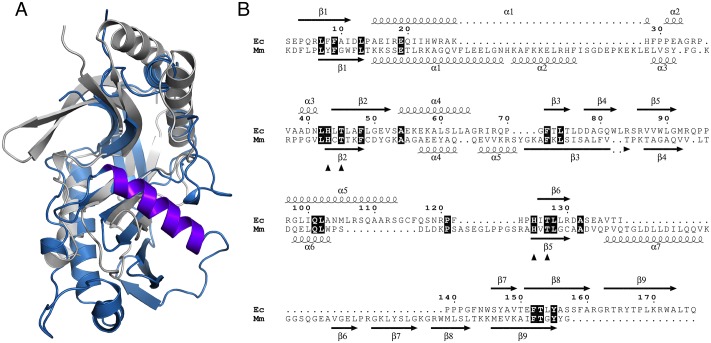
Comparison of CNPase and LigT. A. Structural superposition of *E*. *coli* LigT (white) and mouse CNPase (blue). Specifically note the unique helix α7 (dark blue) in CNPase, lining the CNPase active site, and blocking access of nucleophiles larger than water. B. Structure-based sequence alignment of LigT (Ec) and CNPase (Mm).

Within the active site region, the presence of helix α7 in CNPase is a major difference (Fig 4A). The CNPase-specific helix α7 is missing in LigT, as predicted based on sequence analysis and a structure-based alignment (Fig 4B). CNPase is the only known 2H phosphoesterase family protein with helix α7 and also the only one, for which the catalytic mechanism has been structurally characterized in detail [5,15–17,19].

### Binding to nucleotides in solution

In light of the X-ray crystallographic results, indicating propensity for binding of different nucleotide compunds, we probed ligand compounds also for binding to LigT in solution, in order to clarify whether the observed ligand complexes were possibly artifacts of the crystallization environment. ITC was carried out for 2′- and 3′-AMP, ATP, and NADP^+^ (S1 Fig). The dissociation constants observed for these compounds were 140, 180, 70, and 40 μM, respectively. In light of the K_m_ values of mouse CNPase towards 2′,3′-cyclic NADP^+^ of around 500 μM [27], the K_d_ values determined for LigT are similar and indicate binding of the studied nucleotide compounds to LigT also in solution, in addition to the crystal lattice.

### Conformational flexibility

The active site is surrounded by several loops, which might provide flexibility in substrate binding. The α5-β6 loop, which emerges from helix α5 and leads to the strand β6 that contains one half of the catalytic residues, is disordered in many structures, but can be resolved in selected monomers in the different crystal forms. In mouse CNPase, this loop corresponds to the mobile loop α6-β5, which may play a role in CNPase interaction with larger substrates. Other flexible loops close to the LigT active site include the C-terminal hairpin loop β8-β9 and the loop connecting strands β4 and β5.

**Fig 5:**
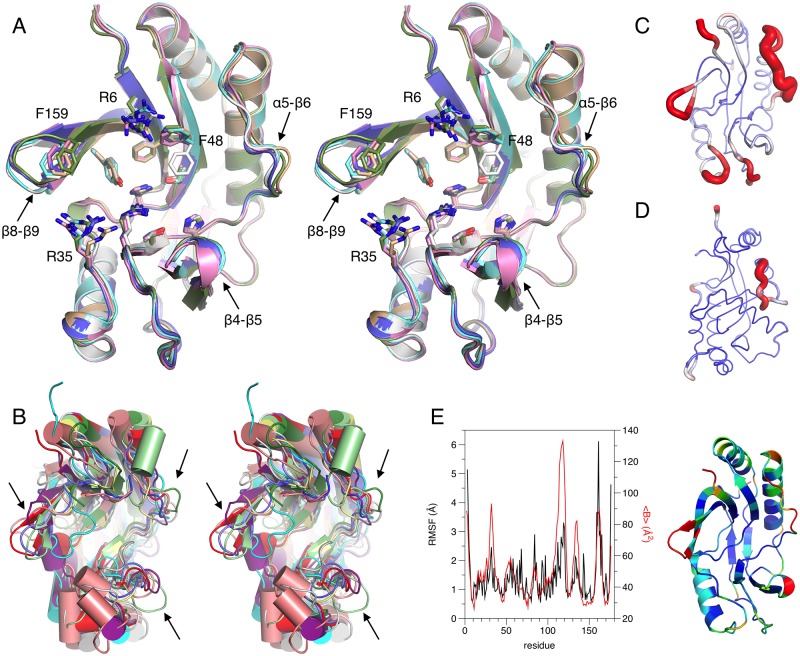
Flexible loops in LigT and the 2H family. A. Superposition of LigT crystal structures in this study (see Fig 3 for colouring details). The three loops marked by arrows present different conformations in the crystal structures; in addition the labeled amino acids differ in conformation between structures. B. Superposition of the structural homologues identified in the Salami search. LigT is red. The 3 flexible loops, showing the largest differences between these 2H enzymes, are highlighted by arrows. Orientation corresponds to the one in A. C. The loops surrounding the active site in LigT show highest *B* factors, *i*.*e*. highest mobility. D. One of the loops also shows specifically high *B* factors in CNPase. E. RMSF (black) of LigT residues during a 260-ns MD simulation and crystallographic *B* factors (red) of LigT (left) and the structure of LigT coloured based on the RMSF (right) indicate that the loops lining the active site are the most flexible segments of the enzyme. Red colour on the structure indicates highest RMSF values.

The LigT ligand complexes highlight mobility of the LigT loops and possible RNA-interacting residues close to the active site. In different crystal forms, the active-site loops can be observed in slightly different conformations (Fig 5A), and especially residues Arg6 and Arg35 take different conformations depending on the bound ligand, further implying their role in phosphomoiety recognition. In a ligand-free active site, also Phe48 is seen to take a conformation distinct from that seen in ligand complexes. Hence, as opposed to CNPase, in which the active site is to a large extent pre-organized for substrate binding [16,17], LigT shows more flexibility.

To get further information on the flexibility of the loops, we performed a search for the closest 2H structural homologues (Fig 5B). The Salami structural homology search detected 11 homologous proteins from the PDB, the best hit representing the previous LigT structure. Vertebrate CNPases were not included in this list, which had 2H proteins from bacteria, viruses, and plants; many of these are annotated as 2′-5′ RNA ligases or phosphoesterases. Superposition of the structures indicated highly similar folding, despite sequence identities as low as 7%, and the largest differences were in the loops mentioned above. This result further highlights the flexibility of the active-site vicinal loops and suggests they may be important in RNA substrate recognition in the entire 2H enzyme family. Visualization of the temperature factors in the LigT crystal structure is in line with this loop flexibility (Fig 5C). The loop corresponding to the α5-β6 loop is also very flexible in mouse CNPase (Fig 5D).

The dynamics of LigT were also studied using MD simulations. Analysis of the root mean square fluctuations (RMSF) during the simulation (Fig 5E) indicates that the most dynamic segments of the protein correspond to the loops described above, surrounding the active-site cavity. The most dynamic structure appears to be the β hairpin formed between the two C-terminal β strands; interestingly, this hairpin loop is not present in the mammalian CNPase structure. Some loops are more rigid in the simulation than predicted by the crystallographic *B* factors; this observation may be related to the fact that the reaction product 2′-AMP was present in the active site in the simulation run. The results further highlight the flexible nature of the loops, likely to play roles in LigT substrate binding.

### Structure in solution

**Fig 6:**
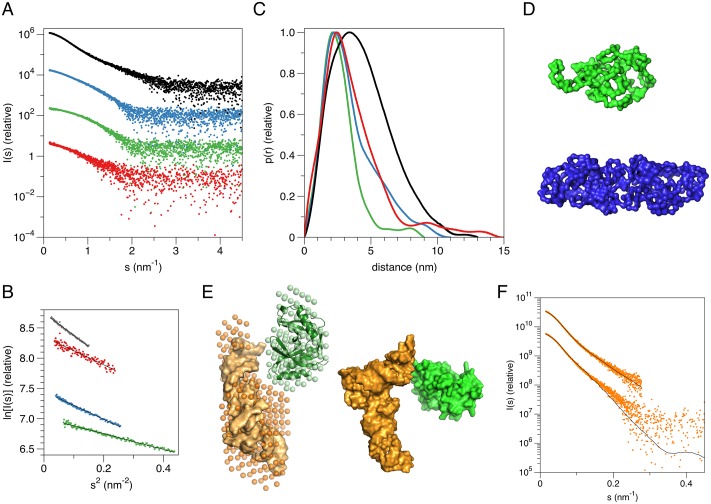
SAXS analysis of LigT. A. Scattering curves of LigT (green), dimeric LigT (blue), yeast tRNA (red), and the LigT-tRNA complex (black). The curves have been vertically displaced for easier viewing. B. Guinier plots of all samples indicate clear lack of aggregation. Plots are shown for 0.5 < sR_g_ < 1.3 for each sample. C. Distance distributions. Note the clear shift of the main peak in the complex sample, indicative of complex formation. D. SAXS-based models of monomeric LigT (top, green) and dimeric LigT (middle, blue). E. Models for the LigT-tRNA complex. Left: The MONSA model is shown as spheres (LigT, green; tRNA, orange), and the crystal structures of LigT and tRNA (PDB entry 2TRA [28]) are superimposed on the model. Right: SASREF rigid body model with the same colouring. Fitting parameters of the models are given in Table 2. F. Fit of the complex models in panel E to the experimental data. Top, MONSA; bottom, SASREF.

To obtain an insight into larger ligand binding, we carried out small-angle X-ray scattering (SAXS) experiments with samples of LigT and yeast tRNA (Fig 6, Table 2). LigT was monomeric in solution, and the obtained 3D shape corresponded closely to that seen in the crystal structure (Chi^2^ = 0.8 between experimental SAXS data and calculated data from a LigT monomer). An exception was the highest concentration (>15 mg/ml), which fitted well to a dimeric species; the relevance of this dimerization is not known, and we believe it was an artifact of the very high concentration. tRNA is slightly larger and more elongated than LigT, as expected. A multiphase *ab initio* modeling approach, taking advantage of the different X-ray scattering properties of RNA and protein, based on SAXS data from the complex and both components alone was employed, and an elongated complex (Fig 6C) fit the experimental data well. The result is a clear indication of the ability of LigT to bind RNA molecules, in this case specifically tRNA. Furthermore, the good fit of the 1:1 complex to the experimental data and the volume of the corresponding *ab initio* model both imply that the sample was mostly in complex form, with free LigT and tRNA not interfering with SAXS analysis. Whether tRNA would be a true biological substrate or ligand for LigT, is unclear at present, and remains a subject for future work. The fact that tRNA was not observed in the crystal structure can be explained by the commercial yeast tRNA preparation being a mixture of different tRNAs, not homogeneous enough to support crystallization of the complex.

**Table 2:** Results from SAXS analysis.

Sample	MW (kDa)	R_g_ (nm)	D_max_ (nm)	Chi^2^
LigT dimer	34.5	2.60 ± 0.01	11	1.8 (GASBOR)
LigT monomer	23	1.98 ± 0.07	9	0.6 (GASBOR)
crystal*	19.8	1.83	5.6	0.8 (CRYSOL)
yeast tRNA	88**	2.66 ± 0.04	15	0.7 (DAMMIN)
LigT-tRNA	129**	3.32 ± 0.04	13	1.1 (MONSA); 2.5 (SASREF)

* values and fits for a monomer crystal structure

** Not reliable, as the MW was determined against a protein standard, and RNA has much higher scattering power than protein. Note, however, that the complex MW corresponds to the sum of the individual components.

### Surface properties for RNA binding

**Fig 7:**
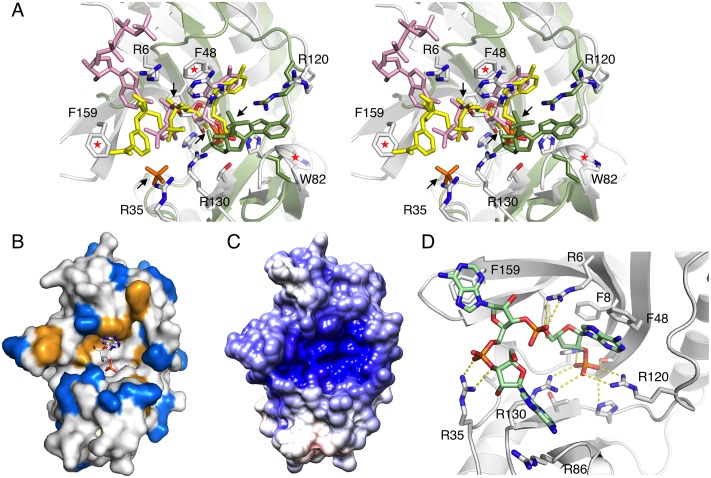
Evidence for an RNA binding surface from individual structures of nucleotide complexes. A. Superimposed are the complexes of LigT (white) with 2′-AMP (white), NADP^+^ (yellow), ATP (pink), and phosphate (orange), as well as the complex of a viral RNase L agonist [29] with adenosine 2′,5′-bisphosphate (green). All the ligands have a phosphate group in the active site, and superposition reveals other nearby features for recognizing phosphate or aromatic base groups in RNA. Arrows indicate the different phosphate binding sites, while red asterisks denote aromatic rings potentially involved in stacking of RNA bases. B. Distribution of aromatic (orange) and basic (blue) groups in LigT. The catalytic site is indicated by the bound reaction product 2′-AMP. C. Surface electrostatic potential of LigT. The catalytic groove has a high positive charge potential. D. Model of binding for a 3-residue RNA molecule with a 2′-phospho group in the active site. Yellow dashed lines indicate the polar contacts by the phosphate moieties. Also pay attention to stacking of bases against Phe8, Phe159, and Arg86. Note Arg120 has flipped over to interact with the active-site phosphate, when the model was energy-minimized and subjected to a short MD simulation.

Some viral and eukaryotic 2H enzymes cleave the 2′-5′-phosphodiester bond of 2′-5′-polyadenylates [11–13]. Structural data [29] from these enzymes incidate that the 2′,5′-adenosine bisphophate substrate binds along the opposite side of the active site, compared to LigT and CNPase (Fig 7A). In CNPase, the side used for 2′,5′-adenosine bisphophate in these enzymes is blocked by the α7 helix, which coordinates the nucleophilic water. In LigT, this opposite side is open, and there would be room for a larger nucleophile than water, even though recent data suggest that short RNA molecules would not act as nucleophiles in LigT [22]. The central phosphate moiety sits nearly identically on top of the HxTx motifs, however. Combining both binding modes by superposition, interesting conservation can be observed (Fig 7A). For example, LigT Trp82 is in a perfect position to stack against a base, and Arg120 is conserved. In the NADP^+^ complex, we actually observe unidentified electron density of a stacking interaction, modeled as a fragment of NADP^+^, on top of Trp82, which could reflect low-affinity binding of some moiety in NADP^+^. These observations highlight several possible sites for interaction between phosphate, sugar, and base moieties in RNA with residues lining the active-site groove in LigT.

LigT has been characterized as an enzyme that can ligate tRNA fragments cleaved by yeast endonuclease. The ligation joins the 2′,3′-cyclic phosphate and 5′-hydroxyl termini in a 2′-5′ phosphodiester linkage, where the linking phosphate group is derived from the cyclic phosphate moiety [10]. Therefore, LigT should bind RNA in the close vicinity of its active site. We looked at the surface properties of LigT to better understand this process.

Earlier, we identified a possible RNA-binding groove in mouse CNPase, which additionally has a polynucleotide kinase-like domain [17]. The surface analysis of LigT, specifically looking at electrostatics, aromatic surface residues, and basic residues, indicates that a similar surface extends away from the LigT active site. The active site is formed at the bottom of a groove lined with basic and aromatic residues, and this groove has a very high positive electrostatic potential (Fig 7B and 7C). These characteristics fit well to the hypothesis of RNA binding.

The substrate of LigT in an RNA ligation reaction is an RNA molecule, for which the 3′-terminal residue, with a 2′,3′-cyclic phosphate group, sits in the active site. The orientation of the reaction products 2′-AMP and NADP^+^ in the active site indicates the direction, into which an RNA molecule would continue, considering the last residue sits in the active site. Likely participants in RNA binding include several aromatic and Arg residues lining the active-site groove.

As the different structures predicted an RNA-binding surface on LigT, we further modeled a 3-residue RNA molecule with a terminal 2′-phosphate into the active site. Possible RNA recognition features can be deduced from this model (Fig 7D), which fit to the binding determinants observed for different ligands crystallographically. The predicted binding mode includes both electrostatic interactions between Arg residues and the phospho groups, stacking of bases against aromatic residues, and C-H…π interactions of the ribose rings.

### The possible functions of LigT

The 2′-5′ phosphodiester linkage between RNA nucleotides is apparently rare, but is nevertheless found all across biology. In animals, 2-5As synthesized by 2′-5′-oligoadenylate synthases have functions in innate immunity, whereby they specifically activate RNase L [30]. RNase L then cleaves singe-stranded viral or cellular RNAs. 2-5As can also have antibacterial activity [31]. Some viruses produce an enzyme that degrades 2′-5′-linked oligoadenylates and prevents the activation of RNase L [13,32]. 2′-5′ oligoadenylates have also been detected in *E*. *coli*. The level of these oligoadenylates increased as a response to phage infection, similarly to animals [20]. Bacterial oligoadenylates are apparently adequate to activate RNase L, since the overexpression of recombinant mammalian nuclease resulted in RNA degradation and cell growth inhibition [33]. LigT might function in the metabolism of bacterial 2-5As [20].

The original paper describing LigT identified it as having enzymatic activity resembling tRNA ligases [10]. These activities included the 3′-phosphodiesterase activity towards the 2′,3′-cyclic phosphate present in the 3′-terminus of the 5′-half of the cleaved tRNA molecule, and the subsequent ligation of the 3′ and 5′ halves with a 2′-5′-phosphodiester bond between the 2′-phosphate group formed in the previous reaction and the 5′-hydroxyl group of the 3′-half of the cleaved tRNA molecule [10]. The ligation was later found to be reversible, as the enzyme additionally functions as a 2′-5′-phosphodiesterase [3,9]. Recently, however, concomitant with the publication of the first *E*. *coli* LigT structure, this view was challenged, as LigT did not appear to ligate short 10-nucleotide RNA oligomers with 2′,3′-cyclic phosphate and 5′-hydroxyl ends, but only acted on the cyclic phosphate [22]. Thus, LigT was claimed to be a CNPase rather that RNA ligase. It should be noted that the experimental conditions in the different studies varied, and it is hard to draw a definite conclusion at this point. Our structural data do highlight close structural similarities of bacterial LigT to RNA ligases and clear differences with respect to the vertebrate CNPase active site.

Despite the current lack of identity of the physiological activity of LigT, the crystal structures presented here further highlight the versatility of the active-site architectures in 2H phosphoesterases. The best-characterized 2H enzyme is mammalian myelin CNPase, for which a central role in the reaction mechanism is played by the N terminus of helix α7 [17]. This helix is missing in most 2H family members, including LigT, and therefore, the reaction mechanisms must also be different across the enzyme family. The α7 helix blocks access of larger nucleophiles than water into the CNPase active site, and it could be argued that its absence in LigT and most other 2H enzymes hints towards larger molecules, most likely RNA, as potential nucleophiles.

## Materials & methods

### Cloning

Genomic DNA from the BL21(DE3) strain of *E*. *coli* was purified and used as a template for PCR (S1 Table). The initial primers for the first PCR added a TEV cleavage site to the N terminus of the coded protein sequence. A second PCR reaction added attB cloning sites to both ends of the insert. The product from the latter reaction was subcloned into the pDONR221 vector (Invitrogen) and further subcloned into the pTH27 expression vector [34], which adds an N-terminal His_6_ tag to the expression product. Clones were verified by DNA sequencing and found to be identical with the LigT database entry (Genbank id. AM946981.2).

### Expression and purification

LigT was overexpressed in the BL21(DE3) strain using ZYM-5052 autoinduction medium [35], supplemented with 100 μg/ml ampicillin and 0.01% Antifoam 204 (Sigma). The expression culture was incubated at +37°C for 24 h. Overexpression did not lead to any obvious toxic effects. The dry cell weight was around 12 g for 1 liter of culture. Cells were harvested by centrifugation and resuspended in lysis buffer containing 50 mM Na-HEPES (pH 7.5), 500 mM NaCl, 20 mM imidazole, 0.5 mM TCEP, and 1x EDTA-free protease inhibitor (Roche). The suspension was flash-frozen in liquid nitrogen and stored at -70°C until use.

The cell suspension was supplemented with 0.1 mg/ml lysozyme and sonicated. The lysate was clarified by a 30-min centrifugation at 27000 g. The clarified lysate was then mixed with Ni-NTA (Qiagen) matrix. The matrix was washed with lysis buffer, and the protein was eluted from the matrix with elution buffer containing 500 mM imidazole. Fractions were analysed using SDS-PAGE. His-tagged TEV protease [36] was added to the eluted fractions containing LigT, in order to remove the His_6_ tag, and this mixture was dialyzed overnight against lysis buffer devoid of imidazole. The dialyzed sample was passed through the Ni-NTA matrix to remove TEV protease, uncleaved LigT, and any other Ni-NTA binding contaminants. The eluted fractions were analyzed using SDS-PAGE. Fractions with cleaved LigT were concentrated and applied to a Superdex 75 16/60 (GE Healthcare) size exclusion chromatography column equilibrated with 10 mM Na-HEPES (pH 7.5), 100 mM NaCl, and 0.5 mM TCEP. Fractions were analysed using SDS-PAGE, and the fractions containing pure LigT were pooled. Approximately 25 mg of pure protein was obtained from one liter of expression culture. Pure LigT was flash-frozen in small batches with liquid nitrogen and stored at -70°C until use.

### Crystallization and data collection

For crystallization, LigT was in the gel filtration buffer at 10 mg/ml. Sitting drop crystallization experiments were prepared with 2:1, 1:1, and 1:2 drop ratios. The crystallization conditions were composed of 0.1 M Tris-HCl at pH 7.4–7.5, 0.2 M MgCl_2_, and PEG 8000 at 12–20% (w/v). For obtaining liganded complexes, LigT was mixed with putative active-site ligands prior to crystallization at 5 mM ligand concentration; these compounds included ATP, NADP^+^, 2′,3′-cAMP, and 3′-AMP. Crystals were cryoprotected by soaking them for a few minutes in the well solution supplemented with the ligand (if present) and 15% (v/v) PEG 200. X-ray diffraction data were collected using synchrotron radiation at 100 K. Data were collected on beamlines P11 and P13 [37] at PETRA III/DESY (Hamburg, Germany), as well as on beamline I911-2 at MAX-Lab (Lund, Sweden). All data were processed using XDS [38].

### Structure solution and refinement

At the time of these experiments, the bacterial LigT structure had not been solved. The structure was solved here by molecular replacement with PHASER [39] using as a search model the LigT orthologue structure from *Thermus thermophilus* [40] (PDB entry 1IUH, sequence identity 28%), modified with phenix.sculptor [41] to resemble more closely the target sequence. Refinement was carried out in phenix.refine [42] and rebuilding in COOT [43]. Final structures were validated using MolProbity [44]. Coordinates and structure factors were deposited at the PDB under accession codes **5LDI** (apo), **5LDJ** (phosphate complex), **5LDK** (ATP complex), **5LDM** (2′-AMP complex), **5LDO** (3′-AMP complex), **5LDP** (ATP complex 2), and **5LDQ** (NADP^+^ complex).

### Structure analysis

Structural sequence alignments were done with Swiss PDB Viewer [45] and Espript [46]. For structure analyses, PyMOL and UCSF Chimera [47] were used. Superpositions were done with the SSM algorithm [48], and structural homologues were searched using Salami [49]. Modeling of the substrate complex and a complex with a 3-base RNA oligonucleotide was done in YASARA [50]. Electrostatic surfaces were calculated using PDB2PQR and ABPS [51].

### Molecular dynamics simulations

The complex of LigT with the bound product 2’-AMP was subjected to MD simulations with the program YASARA [50], version 16.2.21. The structure was placed in a cubic box filled with water, the pH was kept at 7.4, and NaCl was added to keep the salt concentration at the physiological 0.9% (w/w). After initial energy minimization, an MD run of 260 ns was carried out at 298 K. The default YASARA settings were used, saving snapshots every 250 ps, and employing the AMBER14 force field. YASARA scripts were further used for simulation data analysis.

### Small-angle X-ray scattering

SAXS data were collected in batch mode on the EMBL/DESY synchrotron beamline P12 [52], using standard procedures [53]. In addition to LigT alone, yeast tRNA (Sigma) and a 1:1 molar mixture of LigT and tRNA were analyzed. Data were processed with the ATSAS package [54]. Distance distribution functions were analyzed using GNOM [55]. For modelling LigT alone, GASBOR [56] was used. For modelling the protein-RNA complex, we used the program MONSA [57] with the protein and RNA in different phases. The tRNA alone was modelled using DAMMIN [57]. Molecular weight was estimated through a comparison of the forward scattering intensity of the sample to that of a fresh sample of monomeric bovine serum albumin.

### Isothermal titration calorimetry

ITC was carried out using an iTC200 instrument (Microcal). In brief, 0.2–0.4 mM LigT was titrated with 5–10 mM ligand (2′-AMP, 3′-AMP, ATP, NADP^+^); both the protein and ligand were in a buffer consisting of 10 mM HEPES (pH 7.5), 0.2 M NaCl, and 0.1 mM TCEP. The titration was carried out at +25°C, and the data were analysed using MicroCal Origin.

## Supporting information

S1 Table Primers used for LigT cloning. (DOCX)

S1 Fig ITC analysis of nucleotide binding by LigT. (PDF)
