# Supplementary material for: Structural aspects of nucleotide ligand binding by a bacterial 2H phosphoesterase
Source: PLoS One. 2017 Jan 31;12(1):e0170355. doi: 10.1371/journal.pone.0170355 (PMC5283653; doi:10.1371/journal.pone.0170355)
Supplement: S1 Table — (DOCX) [file pone.0170355.s001.docx]

**Table S1.** Primers used for LigT cloning.

| Primer |  |
| --- | --- |
| Forward | GCTCTGAGAATCTTTATTTTCAGGGCatgtctgaaccgcaacgt |
| Reverse | AGAAAGCTGGGTttattgcgtgagcgccca |
| attB-forward | GGGGACAAGTTTGTACAAAAAAGCAGGCTCTGAGAATC |
| attB-reverse | GGGGACCACTTTGTACAAGAAAGCTGGGT |
|  |  |
| Sequence | ATGTCTGAACCGCAACGTCTGTTCTTTGCTATCGACTTACCTGCAGAAATCCGCGAACAGATTATCCACTGGCGCGCCAAACACTTCCCACCTGAGGCGGGACGTCCGGTCGCCGCCGATAATTTGCATCTGACTCTGGCATTTTTAGGCGAAGTGAGCGCCGAGAAAGAGAAGGCGCTTTCTCTTTTAGCCGGACGGATTCGTCAACCTGGTTTCACACTCACGCTTGATGACGCCGGACAATGGCTGCGTTCGCGTGTGGTGTGGTTAGGGATGCGTCAGCCTCCACGCGGCTTAATCCAGCTGGCGAATATGCTCCGTTCACAGGCTGCCCGCAGCGGTTGTTTTCAAAGCAATCGTCCGTTTCATCCACATATTACCTTATTGCGCGACGCCAGCGAGGCGGTGACAATCCCGCCGCCGGGTTTTAACTGGTCTTATGCGGTGACGGAGTTCACCCTTTACGCCTCCTCGTTTGCCCGTGGACGCACACGCTACACGCCGCTAAAACGCTGGGCGCTCACGCAATAA |
