# Supplementary material for: Structural aspects of nucleotide ligand binding by a bacterial 2H phosphoesterase
Source: PLoS One. 2017 Jan 31;12(1):e0170355. doi: 10.1371/journal.pone.0170355 (PMC5283653; doi:10.1371/journal.pone.0170355)
Supplement: S1 Fig — (PDF) [file pone.0170355.s002.pdf]

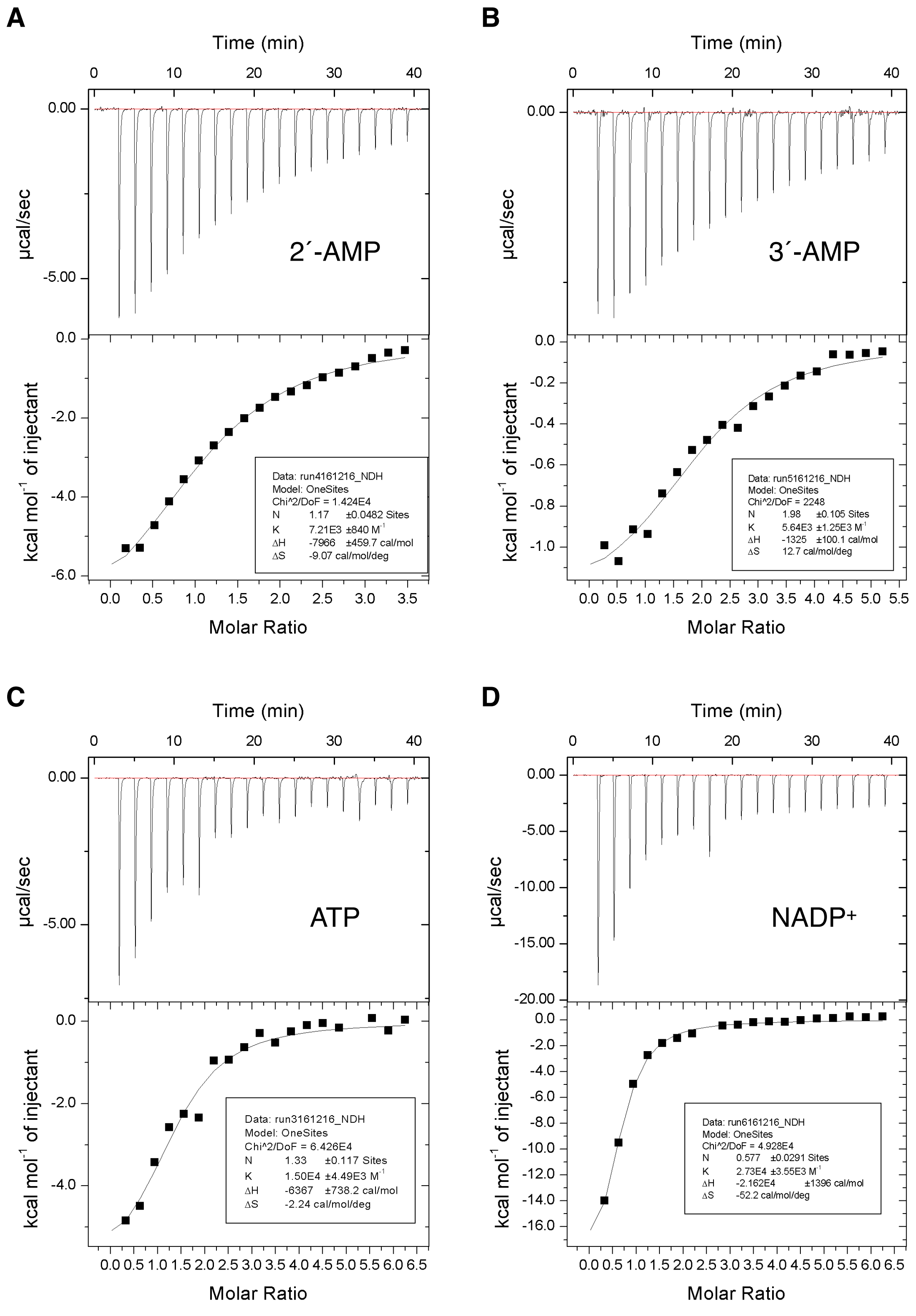

Supplementary Figure 1. Calorimetric binding assays for LigT and 2´-AMP (A), 3´-AMP (B), ATP (C), and $NADP^+$ (D).
